# Green tea's impact on fertility hormones and oxidative stress markers in obese males with different gonadal statuses in Onitsha, Nigeria

**DOI:** 10.4314/gmj.v59i4.8

**Published:** 2025-12

**Authors:** Ifeoma J Onuora, Samuel C Meludu, Chikaodili N Obi-Ezeani, Emmanuel C Dioka, Obiageli E Nnoruka

**Affiliations:** 1 Department of Medical Laboratory Science, Chukwuemeka Odumegwu Ojukwu University, Igbariam, Anambra State, Nigeria; 2 Department of Human Biochemistry, Nnamdi Azikiwe University, Awka, Anambra State, Nigeria; 3 Department of Chemical Pathology, Nnamdi Azikiwe University, Awka, Anambra State, Nigeria

**Keywords:** Obesity, Green tea, Fertility hormones, Oxidative stress, Gonadal dysfunction

## Abstract

**Objectives:**

This study assessed the effects of 12-week green tea supplementation on fertility hormones and oxidative stress markers in obese males with varying gonadal statuses.

**Design:**

A 12-week interventional study measuring hormonal and oxidative stress changes pre- and post-supplementation.

**Setting:**

Conducted in Onitsha, Anambra State, involving community-dwelling obese males categorised by gonadal status.

**Participants:**

One hundred obese males (aged 29–50) were categorised into Eugonadism, Compensatory Hypogonadism, and Hypergonadotropic Hypogonadism groups. Normal-weight eugonadic males served as controls. Participants were selected based on BMI and gonadal hormone levels.

**Interventions:**

Participants consumed two bags of green tea infused in 150 mL of boiled water daily for 12 weeks. Blood samples were collected at baseline, 1 month, and 2 months. Hormonal levels: luteinising hormone (LH), Follicle Stimulating Hormone (FSH), prolactin, testosterone, and oestradiol were measured using ELISA, while oxidative stress markers: malondialdehyde (MDA), total antioxidant capacity (TAC), and glutathione peroxidase (GPx) were analysed colourimetrically.

**Main outcome measures:**

changes in oxidative stress markers and reproductive hormone levels post-supplementation.

**Results:**

Obese participants had significantly higher MDA and lower TAC and GPx than controls (p<0.05). Green tea significantly reduced MDA, oestradiol, and prolactin, whereas it increased TAC, GPx, and testosterone (p < 0.05). LH and FSH were normalised in Eugonadic and Compensatory Hypogonadic groups but remained elevated in Hypergonadotropic Hypogonadism.

**Conclusions:**

Green tea supplementation reduced oxidative stress and improved hormonal balance in obese males, suggesting its potential for managing obesity-related reproductive dysfunction. Further studies should explore long-term effects.

**Funding:**

## Introduction

Obesity is a global health challenge characterised by excessive fat accumulation that adversely affects health.^1^ The prevalence of obesity has risen sharply over recent decades, with profound implications for public health due to its association with chronic diseases such as cardiovascular disorders, diabetes, and metabolic syndrome.^2^ Beyond these widely recognised consequences, obesity has been linked to reproductive dysfunction in men, including alterations in fertility hormones and increased oxidative stress.^3^

Male reproductive health is critically influenced by hormonal balance. Testosterone, luteinizing hormone (LH), and follicle-stimulating hormone (FSH) play pivotal roles in spermatogenesis and overall fertility.

Studies have demonstrated that obesity disrupts the hypothalamic-pituitary-gonadal (HPG) axis, leading to reduced testosterone levels and impairing reproductive function.^4^ Furthermore, obesity-induced oxidative stress—an imbalance between the production of reactive oxygen species (ROS) and the body's antioxidant defenses—has been implicated in sperm dysfunction and DNA damage, further compromising fertility.^5^

Green tea (Camellia sinensis) is widely consumed for its health benefits, attributed to its high content of bioactive polyphenols, particularly catechins such as epigallocatechin gallate (EGCG). These compounds exhibit potent antioxidant properties and have been reported to mitigate oxidative stress and improve metabolic health.^6^ In animal models, green tea supplementation has been shown to ameliorate obesity-related hormonal imbalances and protect against oxidative damage.^7^,^8^ Despite these promising findings, there is limited research on the effects of green tea supplementation on fertility hormones and oxidative stress markers in obese human males with varying gonadal statuses.

This study aims to investigate the impact of green tea supplementation on fertility hormones and oxidative stress markers in obese male subjects with different gonadal statuses. By elucidating the potential therapeutic effects of green tea, this research seeks to contribute to the growing body of evidence supporting dietary interventions for improving male reproductive health. The findings may have implications for developing cost-effective strategies to mitigate the adverse effects of obesity on fertility.

## Methods

### Subject/Study Design

This study employed a cross-sectional design involving 100 male participants: 50 obese and 50 normal-weight controls. The sample size was determined using the formula for comparing two independent means, with 80% statistical power and a significance level of 5%, based on previously published data on oxidative stress markers in obese versus normal-weight males.^2^,^3^ The calculation yielded a minimum of 45 participants per group; however, to strengthen statistical power and account for potential dropouts, 50 participants were recruited into each group.

All participants underwent baseline screening of reproductive hormone levels (LH, FSH, and testosterone) to classify gonadal status. Classification was based on the criteria of Rey et al. (2012):^9^

- **Eugonadism:** Normal LH, FSH, and testosterone levels
- **Compensated Hypogonadism:** Elevated LH and/or FSH with normal testosterone
- **Hypergonadotropic Hypogonadism:** Elevated LH and FSH with low testosterone

Among the obese group, 17 (34%) were Eugonadic, 12 (24%) had Compensated Hypogonadism, and 11 (22%) had Hypergonadotropic Hypogonadism. For the intervention phase, 30 obese participants were randomly selected—10 from each gonadal category (Groups A–C). In addition, 10 normal-weight Eugonadic participants were randomly selected as controls (Group D).

### Randomisation Procedure

Within each gonadal subgroup, simple randomisation was applied. The names of eligible participants were coded and entered into a computer-based random number generator (Research Randomizer®, version 20). The first 10 unique numbers generated in each category were selected to form Groups A–C. Similarly, 10 control participants were randomly selected from the normal-weight pool to form Group D. This approach ensured unbiased selection and equal allocation across groups.

### Constituted Groups

- **Group A:** Eugonadic Obese (n = 10)
- **Group B:** Compensated Hypogonadism Obese (n = 10)
- **Group C:** Hypergonadotropic Hypogonadism Obese (n = 10)
- **Group D:** Eugonadic Normal-Weight Controls (n = 10)

Normal reference intervals for reproductive hormones were defined as follows: LH = 0.7–7.4 mIU/mL; FSH = 1–14 mIU/mL; Prolactin = 1.8–17 ng/mL; Oestradiol = 4–94 pg/mL; Testosterone = 2.5–10 ng/mL.

### Green Tea Supplementation

The green tea was obtained from Lipton Company (Unilever Nigeria Ltd) and was of the same brand and batch number 16252. NAFDAC Reg. NO: B1-8866. Phytochemical analysis of green tea was carried out using gas chromatography to determine the concentration of active ingredients (phenols).

EGCG (the most active green tea catechin) contains 63.4mg/g of the green tea leaves. Two green tea bags weigh 3.2g of green tea leaves. Therefore, two bags of green tea contain 202.9mg of EGCG, which is within the recommended mean daily intake of 90–300mg/day for effective biological action, according to the European Food Safety Authority.^10^

Phytochemical content of Commercial Green Tea Supplement (Lipton/Unilever Product) per sachet (1.6g) was:

**Table:** 

POLYPHENOLS	CONCENTRATION (mg/g)
**caffeine**	24
**Epicatechin (EC)**	14.3
**Epigallocatechin (EGC)**	76.3
**Epicatechin gallate (ECG)**	8.2
**Epigallocatechin gallate (EGCG)**	63.4
**Catechin (C)**	2.6
**Tannin**	1.7
**Saponin**	1.27
**Oxalate**	2.2
**TOTAL**	**165mg/g**

### Green Tea Preparation

Two (2) green tea bags, each weighing 1.6g, were soaked in 150ml of boiled water for 5 minutes before consumption. The green tea was taken once daily according to the manufacturer's instructions for 12 weeks.^11^ To ensure adherence to green tea supplementation instructions, participants were contacted by telephone twice weekly throughout the 8-week intervention. During each call, the research assistant confirmed daily consumption of the assigned green tea dose, reminded participants of the timing and method of intake, and enquired about any adverse effects. Compliance was assessed using a structured checklist, and participants were encouraged to return unused sachets at the next clinic visit for verification.

Telephone follow-up significantly improved compliance: 92% of participants reported full adherence to supplementation instructions, while 8% missed one or more doses. Cross-checking of returned sachets at clinic visits confirmed these self-reports, indicating that the follow-up strategy effectively addressed adherence concerns.

### Inclusion Criteria / Exclusion Criteria

Apparently healthy obese and normal weight male subjects between the ages of 29 and 50 years with body mass index 30 – 39.9 Kg/m2 and 18.5 -24.9, respectively, residing in Onitsha were enrolled. Morbidly obese (BMI above 41Kg/m^2^), and participants above 50 years, who smoked cigarettes, drank alcohol and used other food supplements were also excluded from the study.

### Ethical Approval

The participants were fully informed about the study design; written informed consent was obtained prior to enrolment. Ethical approval was sought and obtained from the Ethics Committee of the Nnamdi Azikiwe University Teaching Hospital (NAUTH), Nnewi, Anambra State, with Reference number:

NAUTH/CS/66/VOL10/2017/010, and it conformed to all the ethical requirements of the Declaration of Helsinki.

#### Anthropometric measurements

The subjects' weights were measured using a scale (Gulfex Medical and Scientific, England). Weight was measured in kilograms (kg) and recorded to the nearest 0.1kg. The subjects' height was recorded in metres using a height scale calibrated in centimetres, and the reading was rounded to the nearest 0.1cm. BMI was calculated as weight (kg) divided by height squared (m^2^). To determine abdominal obesity, waist circumference (WC) was measured using a stretch-resistant tape (HTS, China).

### Sample Collection

Following an overnight fast (10–12 hours), 5 mL of venous blood was collected aseptically from each participant using a sterile disposable syringe. Samples were obtained at baseline (prior to green tea supplementation) and subsequently at 6 and 12 weeks of the intervention. Blood was dispensed into plain vacutainer tubes, allowed to clot at room temperature, and centrifuged at 3,000 rpm for 10 min to obtain serum. The serum was aliquoted into labelled cryovials and stored at −20 °C until analysis. All biochemical assays were performed within three months of collection to preserve sample integrity.

Serum aliquots were analysed for:

- **Oxidative stress markers:** total antioxidant capacity (TAC), malondialdehyde (MDA), superoxide dismutase (SOD), catalase (CAT), and glutathione peroxidase (GPx), using standard spectrophotometric methods.
- **Hormonal assays:** luteinizing hormone (LH), follicle-stimulating hormone (FSH), prolactin, testosterone, and oestradiol, determined using enzymelinked immunosorbent assay (ELISA) kits.

### Biochemical Analyses

Serum Luteinizing hormone (LH), Follicular Stimulating Hormone (FSH), Testosterone, Oestradiol, and prolactin levels were measured by solid-phase enzyme-linked immunosorbent assay (ELISA) using the ACUBIND kit and the Mindray (MR-96A) ELISA machine. MDA, TAC and GPx were assayed colourimetrically. Pooled control sera from apparently healthy individuals were used to control the assay.

### Statistical analyses

The data were analysed using the Statistical Package for the Social Sciences (SPSS) version 23. The variables were expressed as mean ± SD. Student's t-test and paired t-test were used to compare the mean difference, with significance set at p < 0.05.

## Results

Participants were classified based on their fertility hormone levels. Out of 50 obese males recruited, 17(34%), 12(24%), and 11(22%) were Eugonadotropic, Compensated Hypogonadotropic and Hypergonadotropic hypogonadic respectively. In Table 1, the results showed that the mean levels of weight, BMI, waist circumference, and MDA were significantly higher, whereas TAC was significantly lower in obese participants than in controls (p < 0.05).

**Table 1:** Mean level of anthropometric and oxidative stress markers in obese and normal weight male subjects. Mean ± SD

PARAMETER	OBESITY	CONTROL	P-VALUE
**AGE (yrs)**	38.6 ± 5.5	36.6 ± 5.0	0.028*
**WEIGHT (kg)**	103 ± 10.5	69.7 ± 4.5	0.001*
**BMI**	35.5 ± 2.8	22.8 ± 1.2	0.001*
**WC (cm)**	110 ± 7.3	84 ± 9.0	0.002*
**MDA (nmol/ml)**	3.8 ± 1.1	3.3 ± 0.87	0.003*
**TAC (µmol/L)**	953.6 ± 177	1063 ± 226	0.002*
**GPx (U/ml)**	1.09 ± 0.19	1.15 ± 0.24	0.095

BMI = Body mass Index, WC = Waist circumference, MDA = Malondealdehyde, TAC= Total Antioxidant Capacity, GPx= Glutathione peroxidase

* Significant at p< 0.05

Furthermore, the mean levels of LH, FSH and testosterone were significantly lower. At the same time, oestradiol and prolactin were significantly higher in obese participants when compared with the control (Normal weight) (p< 0.05), as shown in Table 2.

**Table 2:** Mean levels of reproductive hormones in obese and normal weight male subjects. Mean ± SD

Parameter	Obesity	Control	P-Value
**LH (mIU/mL)**	4.8 ± 1.5	5.4 ± 1.9	0.038*
**FSH (mIU/mL)**	4.5 ± 1.4	5.6 ± 1.9	0.001*
**PROLACTIN (ng/mL)**	8.2 ± 7.0	7.0 ± 2.6	0.008*
**TESTOSTERONE (ng/mL)**	6.02 ± 1.9	7.5 ± 2.2	0.002*
**OESTRADIOL (pg/mL)**	104 ± 20	83.7 ± 11	0.001*

* Significant at p< 0.05 LH= Leutenising Hormones, FSH = Follicular Stimulating Hormones

Levels of oxidative stress markers among groups classified by gonadal status showed that the Eugonadic, Compensatory Hypogonadism, and Hypergonadotrophic Hypogonadism Obese groups had significantly higher levels of MDA and lower levels of TAC and GPx than the Eugonadic normal-weight (control) group (p<0.05).

Testosterone and oestradiol were also significantly higher in Eugonadic Obese, Compensatory Hypogonadic Obese and Eugonadic Control groups when compared with Hypergonadotropic Hypogonadic Obese Participants (p<0.05). LH and FSH were significantly lower (p<0.05) when comparing Eugonadic obese with Compensatory Hypogonadic Obese, as shown in Table 3. Following 12 weeks of green tea supplementation, there were significant increases in the mean levels of LH, FSH, TAC, and GPx, and a decrease in MDA, oestradiol, prolactin, weight, and waist circumference in Eugonadal obese participants compared with baseline (p<0.05). Testosterone level did not increase significantly when compared with baseline (p>0.05). All the studied hormonal values were still within the normal reference range as shown in Table 4.

**Table 3:** Comparison of oxidative stress markers and fertility hormones among groups classified based on gonadal status

Parameter	Group A	Group B	Group C	Group D	A vs B	P-value Avs C	P-value Avs D	P-value BvsC	P-value Bvs D	P-value-CvsD
**Oxidative Stress Markers**										
**MDAnmol/mL**	3.6 ± 1.2	4.7 ± 0.38	5.1 ± .71	3.0±0.78	0.016*	<.001*	0.005*	<.001*	<.001*	<.001*
**TAC (µmol/L)**	957±183	956 ± 149	914± 108	1061±152	1.000	1.000	0.002*	1.000	0.002*	<.001*
**GPX (U/mL)**	1.08 ± 0.16	1.0 ± 0.06	1.1 ±0.34	1.1±0.24	0.866	1.000	0.008*	0.466	0.012*	0.009*
**Fertility Hormones**										
**LH (mIU/mL)**	4.4 ± 1.6	9.2 ± 1.1	9.8 ± 1.9	5.4 ± 1.7	<.001*	<.001*	0.029*	1.000	<.001*	<.001*
**FSH (mIU/mL)**	3.8 ± 1.3	14.8 ± 0.87	15.5 ± 4.4	5.1 ± 1.7	<.001*	<.001*	0.008*	1.000	<.001*	<.001*
**Prolactin(ng/mL)**	9.4 ± 4.5	8.6 ± 4.0	13 ± 5.3	7.5 ± 3.1	1.000	0.042*	<.042*	0.244	1.000	<.001*
**Testosterone(ng/mL)**	5.7 ± 2.2	3.2 ± 1.1	1.5 ± 0.65	6.3 ± 1.7	<.001*	<.001*	1.000	<.001*	0.412	<.001*
**Oestradiol(pg/mL)**	100 ± 15.8	75 ± 18.7	28 ± 5.8	84.22 ±11	<.001*	<.001*	<.001*	<.001*	<.001*	<.001*

Values are in mean ± SD, and p<0.05 is considered statistically significant. Group A= Eugonadic obese, Group B= Compensatory hypogonadism obese, Group C= Hypergonadotrophic hypogonadism group, Group D= Eugonadic normal weight.

Normal Reference interval for Hormones LH= 0-7.4(mIU/mL), FSH 1-14(mIU/mL), prolactin 1.8- 17(ng/mL), Testosterone 2.5-10(ng/mL), Oestradiol 4-94(pg/mL)

**Table 4:** Effect of green tea on anthropometric parameters, male reproductive hormones and oxidative stress markers of eugonadic male obese participants at different stages of green tea supplementation

PARAMETERS	BASELINE (A)	6-WEEKS (B)	12-WEEKS (C)	P-value A vs B	P-value A vs C
**Weight (Kg)**	104 ± 9	98.4 ± 8.5	94.2 ± 2.2	0.098	0.007*
**WC (cm)**	112.1 ± 6.3	104.2 ± 5.2	99.8 ± 7.0	0.015*	0.001*
**MDA(nmol/ml)**	4.2 ± 0.38	3.9 ± 1.2	3.2 ± 1.1	0.417	0.036*
**TAC (µmol/L)**	933 ± 152.8	981 ± 154	1276 ± 62	0.399	0.001*
**GPx (U/ml)**	1.24 ± 0.27	1.13 ± 0.14	1.04 ± 0.11	0.081	0.047*
**LH (mIU/mL)**	4.59 ± 0.5	4.8 ± 0.80	5.2 ± 0.80	0.486	0.014*
**FSH (mIU/mL)**	4.27 ± 1.	4.8 ± 0.7	5.3 ± 1.2	0.073	0.010*
**Prolactin (ng/mL)**	9.0 ± 1.7	7.3 ± 1.	7.0 ± 1.5	0.022*	0.024*
**Testosterone(ng/mL)**	5.5 ± 1.8	5.8 ± 1.9	6.2 ± 1.5	0.695	0.429
**Oestradiol (pg/mL)**	104 ± 10.3	97 ± 9.1	94 ± 8.8	0.158	0.010*

* Significant at p<0.05

Eugonadism = Normal LH, FSH and Testosterone

In Table 5, there were significant increases in the mean level of TAC, testosterone, and a significant decrease in LH, FSH, MDA, prolactin, weight and waist circumference in Compensatory Hypogonadic obese participants when compared with baseline (p<0.05) after 12-week green tea supplementation. The mean levels of gonadotrophic hormones (LH and FSH) reduced significantly and fell within the normal reference range. In Table 6, there were significant increases in the mean level of TAC and testosterone and a significant decrease in LH, FSH, MDA, prolactin, oestradiol, weight and waist circumference in Hypergonadotropic Hypogonadic obese participants when compared with baseline (p<0.05) after 12-week green tea supplementation. The mean levels of gonadotrophic hormones (LH and FSH), though reduced significantly, did not fall within the normal reference range.

**Table 5:** Effect of green tea on male reproductive hormones and oxidative stress markers of compensatory hypogonadic male obese participants at different stages of green tea supplementation

PARAMETERS	BASELINE	6-WEEKS	12-WEEKS	P-value	P-Value
N=10	(A)	(B)	(C)	A vs B	A vs C
**Weight (Kg)**	110.40±7.4	110.20 ± 7.7	107 ± 8.0	0.555	0.047*
**WC (cm)**	120.3 ± 2.9	120.1 ± 3.5	113 ± .40	0.509	0.002*
**MDA(nmol/ml)**	4.20 ± 0.70	3.77 ± 0.76	3.42 ± 0.56	0.154	0.003*
**TAC (µmol/L)**	921.3 ± 158	959 ± 176	1261.2 ± 172	0.314	0.001*
**GPx (U/ml)**	0.99 ± 0.14	1.14 ± 0.15	1.15 ± 0.21	0.095	0.090
**LH (mIU/mL)**	10.4 ± 1.4	8.7 ± 1.0	6.8 ± 0.78	0.013*	0.031*
**FSH (mIU/mL)**	14.7 ± 0.48	11.4 ± 3.2	10.4 ± 0.67	0.134	<0.001*
**Prolactin (ng/mL)**	7.4 ± 0.49	7.1 ± 0.66	6.4 ± 0.79	0.094	0.007*
**Testosterone(ng/mL)**	3.9 ± 1.0	6.7 ± 0.71	8.6 ± 0.48	0.107	0.020*
**Oestradiol (pg/mL)**	100 ± 20	90 ± 10.7	80 ± 9.9	0.202	0.076

* Significant p<0.05

Compensatory Hypogonadism = High LH, FSH and Normal Testosterone serum level

**Table 6:** Effect of green tea on male reproductive hormones and oxidative stress markers of hypergonadotropic hypogonadic male obese participants at different stages of green tea supplementation

PARAMETERS N=10	BASELINE (A)	6-WEEKS (B)	12-WEEKS (C)	P-VALUE A vs B	P-VALUE A vs C
	119.9 ± 11	109 ± 7.1	102 ± 12.6	0.108	0.014*
**WC (cm)**	122 ± 4.1	121.5 ± 4.0	114.6 ± 5.2	0.779	0.016*
**MDA(nmol/ml)**	4.5 ± 0.62	4.0 ± 0.69	3.8 ± 0.40	0.093	0.017*
**TAC (µmol/L)**	886 ± 102	906 ± 78	1162 ± 172	0.552	0.003*
**GPx (U/ml)**	0.97 ± 0.14	1.01 ± 0.19	1.05 ± 0.18	0.420	0.214
**LH (mIU/mL)**	10.9 ± 2.0	9.3 ± 0.83	8.0 ± 0.51	0.126	0.006*
**FSH (mIU/mL)**	16.7 ± 2.2	16.0. ± 2.3	14.9 ± 2.1	0.221	0.017*
**Prolactin (ng/mL)**	13.7 ± 5.6	11.2 ± 4.5	10 ± 3.8	0.024*	0.032*
**Testosterone(ng/mL)**	1.2 ± 0.68	3.3 ± 0.15	3.8 ± 0.29	0.012*	0.008*
**Oestradiol (pg/mL)**	40 ± 2.5	37 ± 2.6	33 ± 3.0	0.030*	0.004*

* Significant p<0.05

Hypergonadotropic hypogonadism = High LH, FSH and Low Testosterone serum level

## Discussion

Obesity is a global health concern associated with adverse effects on male reproductive health, including hormonal imbalances and oxidative stress. The results of this study indicate that the mean levels of weight, BMI, waist circumference, and MDA were significantly higher, while TAC was significantly lower in obese participants compared with controls (normal weight) (p < 0.05).

These findings are consistent with previous studies that reported increased oxidative stress and altered antioxidant defense mechanisms among obese individuals, confirming the close association between adiposity and oxidative imbalance. 2,8,12

In addition, the hormonal analysis revealed significantly lower levels of LH, FSH, and testosterone, alongside higher oestradiol and prolactin levels in obese participants compared to controls (p < 0.05). These findings align with prior research illustrating the adverse effects of obesity on the hypothalamic-pituitary-gonadal axis.^4^ MacDonald *et al.* conducted a meta-analysis revealing that obesity is associated with reduced testosterone and gonadotropin levels, likely due to increased aromatization of androgens to estrogens in adipose tissue.^4^ Elevated oestradiol levels, in turn, suppress gonadotropin release through negative feedback mechanisms, further exacerbating hormonal imbalances.

The 12-week green tea supplementation yielded significant improvements in oxidative stress markers and hormonal profiles across the study groups, although effects varied by gonadal status. In Eugonadic obese participants, supplementation resulted in significant increases in LH, FSH, TAC, and GPx, along with decreases in MDA, oestradiol, prolactin, weight, and waist circumference (p < 0.05). These findings align with the work of Khan and Mukhtar who demonstrated the antioxidative potential of green tea catechins in reducing lipid peroxidation and improving hormonal balance.^7^ However, the lack of a significant increase in testosterone levels (p > 0.05) may indicate a threshold effect, where moderate hormonal disruptions in Eugonadic obese participants are insufficiently severe to elicit notable testosterone changes post-intervention.

Similarly, Compensatory Hypogonadic obese participants exhibited significant increases in TAC, testosterone, and GPx, alongside reductions in LH, FSH, MDA, prolactin, weight, and waist circumference (p < 0.05). The observed normalisation of gonadotrophis hormones (LH and FSH) within reference ranges highlights the modulatory effects of green tea catechins on the HPG axis. These results are consistent with findings by Chavarro et al., who reported that antioxidant-rich diets positively impact gonadal function by mitigating oxidative stress and inflammatory pathways.^13^

The reductions in weight and waist circumference observed in both groups further underscore the metabolic benefits of green tea supplementation, particularly for individuals consuming green tea catechins.^2^ Weight reduction likely contributed to decreased oestradiol levels by reducing adipose tissue aromatase activity, thereby mitigating androgen-to-estrogen conversion.^14^ Concurrent decreases in prolactin levels may reflect improved metabolic and endocrine homeostasis, as prolactin dysregulation is closely linked to obesity and insulin resistance.^15^

In contrast to Eugonadic obese participants, the Compensatory Hypogonadic group showed a significant increase in testosterone levels (p < 0.05), suggesting that individuals with more pronounced gonadal dysfunction may exhibit greater responsiveness to green tea's antioxidative and anti-inflammatory effects. This aligns with findings by Aoki et al., who observed enhanced testosterone biosynthesis in hypogonadic models supplemented with green tea extracts.^16^

In Hypergonadotropic Hypogonadic obese participants, supplementation resulted in significant increases in TAC and testosterone levels and reductions in LH, FSH, MDA, prolactin, oestradiol, weight, and waist circumference (p < 0.05). Although gonadotrophic hormone (LH and FSH) levels decreased significantly, they did not normalise, indicating persistent dysregulation of the HPG axis despite intervention. This finding contrasts with observations in Eugonadic and Compensatory Hypogonadic groups, where gonadotrophic hormone levels either normalised or showed greater responsiveness to green tea catechins. This discrepancy may reflect the severity of gonadal dysfunction in Hypergonadotropic Hypogonadic participants, as advanced dysfunction often entails irreversible damage to testicular Leydig and Sertoli cells.^17^

This study has some limitations that should be acknowledged. First, the sample size was relatively small, which may limit the statistical power and generalisability of the findings. Second, only male participants were included, making it difficult to extrapolate the results to females or mixed populations. Third, the intervention period was relatively short, and the long-term effects of green tea supplementation on oxidative stress and hormonal status were not assessed. In addition, adherence was primarily monitored through self-report and telephone follow-up, which may be subject to reporting bias despite sachet cross-checking. Finally, the study was conducted in a single geographical location, and dietary or lifestyle factors not fully controlled for may have influenced the outcomes. Future studies should examine the long-term effects of supplementation and the underlying molecular mechanisms to refine its application in clinical settings.

## Conclusion

Green tea supplementation significantly improved anti-oxidant status and modulated hormonal indices across the different gonadal categories. These findings suggest that *Camellia sinensis* may have therapeutic potential in mitigating oxidative stress and supporting reproductive hormonal balance in obese males.
